# Achieving Negatively Charged Pt Single Atoms on Amorphous Ni(OH)_2_ Nanosheets with Promoted Hydrogen Absorption in Hydrogen Evolution

**DOI:** 10.1007/s40820-024-01420-6

**Published:** 2024-05-23

**Authors:** Yue Liu, Gui Liu, Xiangyu Chen, Chuang Xue, Mingke Sun, Yifei Liu, Jianxin Kang, Xiujuan Sun, Lin Guo

**Affiliations:** 1 School of Chemistry, Beijing Advanced Innovation Center for Biomedical Engineering, Key Laboratory of Bio-inspired Smart Interfacial Science and Technology, Beihang University, Beijing, 100191 People’s Republic of China; 2 School of Chemistry, Key Laboratory of Environmentally Friendly Chemistry and Applications of Ministry of Education, Xiangtan University, Xiangtan, 411105 Hunan People’s Republic of China

**Keywords:** Hydrogen evolution reaction, Amorphous, Pt single atoms, Hydrogen spillover

## Abstract

Single-atom (SA) catalysts with nearly 100% atom utilization have been widely employed in electrolysis for decades, due to the outperforming catalytic activity and selectivity. However, most of the reported SA catalysts are fixed through the strong bonding between the dispersed single metallic atoms with nonmetallic atoms of the substrates, which greatly limits the controllable regulation of electrocatalytic activity of SA catalysts. In this work, Pt–Ni bonded Pt SA catalyst with adjustable electronic states was successfully constructed through a controllable electrochemical reduction on the coordination unsaturated amorphous Ni(OH)_2_ nanosheet arrays. Based on the X-ray absorption fine structure analysis and first-principles calculations, Pt SA was bonded with Ni sites of amorphous Ni(OH)_2_, rather than conventional O sites, resulting in negatively charged Pt^*δ*−^. In situ Raman spectroscopy revealed that the changed configuration and electronic states greatly enhanced absorbability for activated hydrogen atoms, which were the essential intermediate for alkaline hydrogen evolution reaction. The hydrogen spillover process was revealed from amorphous Ni(OH)_2_ that effectively cleave the H–O–H bond of H_2_O and produce H atom to the Pt SA sites, leading to a low overpotential of 48 mV in alkaline electrolyte at −1000 mA cm^−2^ mg^−1^_Pt_, evidently better than commercial Pt/C catalysts. This work provided new strategy for the controllable modulation of the local structure of SA catalysts and the systematic regulation of the electronic states.

**Supplementary Information:**

The online version contains supplementary material available at 10.1007/s40820-024-01420-6.

## Highlights

- Pt–Ni bonded Pt single-atom (SA) catalyst, rather than classic Pt–O bonded SA catalyst, was successfully constructed.
- The electronic states of Pt SA catalyst were deeply regulated and negatively charged Pt^*δ*−^ was realized.
- Pt–Ni bonded Pt SA catalyst-enhanced absorbability for activated hydrogen atoms and promoted hydrogen absorption.

**Supplementary Information:**

The online version contains supplementary material available at 10.1007/s40820-024-01420-6.

## Graphical abstract

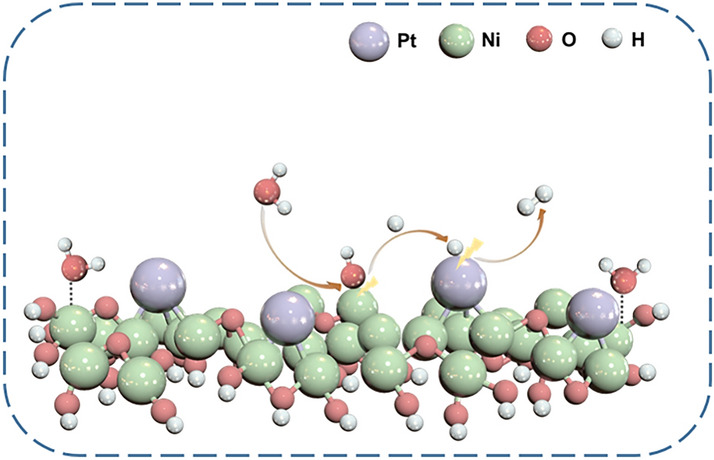

## Introduction

Hydrogen evolution reaction (HER), as a half-reaction of water electrolysis, provides a practical way to produce hydrogen with excellent energy conversion efficiency, arousing great interest in the exploration and development of highly active catalysts [1–4]. Among all the catalysts, noble metal-based catalysts represented by Pt demonstrate excellent specific activity and selectivity, which are widely used in HER [5–7]. As the scarce reserves and expensive prices seriously limit their scaling up, single-atom (SA) catalysts with the maximum atomic utilization, afford an alternative for the application of noble metal-based catalysts [8–10]. However, in order to fix the metal atoms and prevent the agglomeration, most of the reported SA catalysts were strongly bonded with the nonmetal atoms (O, N, etc.) of the substrate [11–13]. It will inevitably lead to the oxidation of the metal atoms and further hinder the regulation of the electronic states, diminishing their electrocatalytic properties. For example, oxidized noble metal atoms would reduce the adsorption capacity for hydrogen atoms, limiting the HER activity of the catalysts [11].

Unlike widely used crystalline substrates, amorphous materials demonstrate advantages as substrates in optimizing the geometrical and electronic structure of SA due to their unique long-range disordered and isotropic structural features [14–19]. The disordered adjacent atoms on the amorphous substrates could geometrically change the active sites and rearrange the distribution of electrons, which may accelerate the charge transfer between active centers, and enhance the electrochemical activity of the active sites [11, 20, 21]. Meanwhile, the abundant dangling bonds and defects in the amorphous substrate can anchor the SA and effectively improve the structural stability of SA catalysts in long-term service [22–24]. The key point is that the highly coordination unsaturated local environment of exposed metal atoms on the surface of amorphous substrate could provide the possibility of constructing metal–metal bonding, instead of the conventional metal-nonmetal bonding for SA loading [7, 8, 25]. The novel structure could subversively modulate the electronic structure of the SA catalysts, further promoting the catalytic performance [20].

Here, we report a novel Pt SA catalyst fixed by Pt–Ni bonding on amorphous Ni(OH)_2_ nanosheet arrays through a controllable electrochemical reduction strategy. The coordination unsaturated atomic structure of amorphous substrate provides sufficient sites for the adsorption of PtCl_6_^2−^ ions, while the nascent Pt SA from reduction is captured by the exposed Ni metal sites at the oxygen vacancies to form Pt–Ni bonds instead of the conventional Pt–O bonds. Both of the density functional theory (DFT) calculation and in situ Raman spectroscopy demonstrate the enhanced absorbability for activated hydrogen atoms, which facilitates hydrogen spillover processes between amorphous Ni(OH)_2_ substrate and Pt SA in the alkaline. The amorphous Ni(OH)_2_ substrate efficiently cleaves water molecules and delivers hydrogen atoms to the Pt sites, significantly increasing the hydrogen coverage on the Pt SA and leading to the enhanced HER performance. This work provides a method for the development of highly active SA catalysts, which could efficiently promote the hydrogen production in alkaline solution.

## Experimental Section

### Chemicals

Carbon fiber paper (TGP-H-060, Toray Industries, Japan), Ni(acac)_2_·2H_2_O (96%, J&K Scientific Inc., China), anisole (99%, Innochem Inc., China), Ni(OH)_2_ (99%, J&K Scientific Inc., China), Pt/C (Pt 20%, Alfa Aesar Inc., USA), Nafion solution (5 wt% in water, J&K Scientific Inc., China), KOH (85%, Alfa Aesar Inc., USA), and chloroplatinic acid (H_2_PtCl_6_) (Pt ≥ 37.5%, Macklin, China).

### Catalysts Preparation

#### Synthesis of Ni(acac)_2_ Nanosheet Arrays

30 mg of Ni(acac)_2_·2H_2_O was placed in a 100 mL flask and 30 mL of anisole was added, followed by a water bath for 1 h to accelerate the dissolution of Ni(acac)_2_·2H_2_O. And then 1 × 2 cm^2^ of carbon fiber paper (CFP) was added to the solution and the flask was moved into an oil bath at 120 °C for 2 h. After that, the CFP was taken out and washed with ethanol several times.

#### Synthesis of a-Ni(OH)_2_ Nanosheet Arrays

The constant current activation method was used to transform Ni(acac)_2_ nanosheet arrays into amorphous Ni(OH)_2_ (a-Ni(OH)_2_) nanosheet arrays by removing the organic groups. In a two-electrode system, the as-prepared nanosheet arrays was employed as the anode and the constant current electrolysis was conducted under a constant current of 5 mA in a 1 M KOH solution for 5–6 h.

#### Synthesis of Pt–SA/a-Ni(OH)_2_

Electroplating was used for the synthesis of single-atom Pt on the a-Ni(OH)_2_ nanosheet arrays (Pt–SA/a-Ni(OH)_2_). In a typical experiment, the a-Ni(OH)_2_ was served as the cathode, together with a carbon rod as the counterpart electrode and Hg/HgO electrode as the reference electrode, and then placed in a 1 M KOH solution containing 10^−5^ M PtCl_6_^2−^ solution. Constant voltage electrolysis was conducted at −0.079 V vs. RHE for 20 min, during which the platinum in the solution is reduced and plated onto the a-Ni(OH)_2_ nanosheet arrays.

#### Synthesis of Pt–NP/a-Ni(OH)_2_

The reference material was synthesis in a similar method with Pt–SA/a-Ni(OH)_2_. For the synthesis of nanoparticle Pt on a-Ni(OH)_2_ nanosheet arrays, the electroplating time was 40 min, with other parameters remaining the same in the synthesis of Pt–SA/a-Ni(OH)_2_.

#### Preparation of Pt/C Catalyst

20 mg of commercial Pt/C (20 wt%) catalyst was dispersed in 990 µL of ethanol, followed by the addition of 10 µL of 5 wt% nafion solution. After 20 min of sonication, a homogeneous ink was obtained. Finally, 100 µL of ink was pipetted to the CFP (1 × 1 cm^2^) on both sides for even coating, and dried in air.

#### Preparation of Pt/CFP

The reference material was synthesized in a similar method with Pt–SA/a-Ni(OH)_2_. For the synthesis of Pt on CFP (Pt/CFP), the substrate for electroplating was the bared carbon fiber paper. And the parameters of electroplating were the same with those in the synthesis of Pt–SA/a-Ni(OH)_2_. In a typical experiment, the loading amount of Pt in Pt/CFP is tested to be 0.15%.

#### Synthesis of Pt–SA/commercial Ni(OH)_2_

For the synthesis of single atom Pt on commercial Ni(OH)_2_ (Pt–SA/commercial Ni(OH)_2_), 5 mg of commercial Ni(OH)_2_ was dispersed in 990 µL of ethanol, followed by the addition of 10 μL of nafion solution. The prepared Ni(OH)_2_ ink was dropped onto a CFP (1 × 2 cm^2^). Next, this CFP was used as the working electrode and the electroplating of single atom Pt was conducted. The parameters of electroplating were the same with those in the synthesis of Pt–SA/a-Ni(OH)_2_.

## Results and Discussion

### Morphology of Pt–SA/a-Ni(OH)_2_

**Fig. 1:**
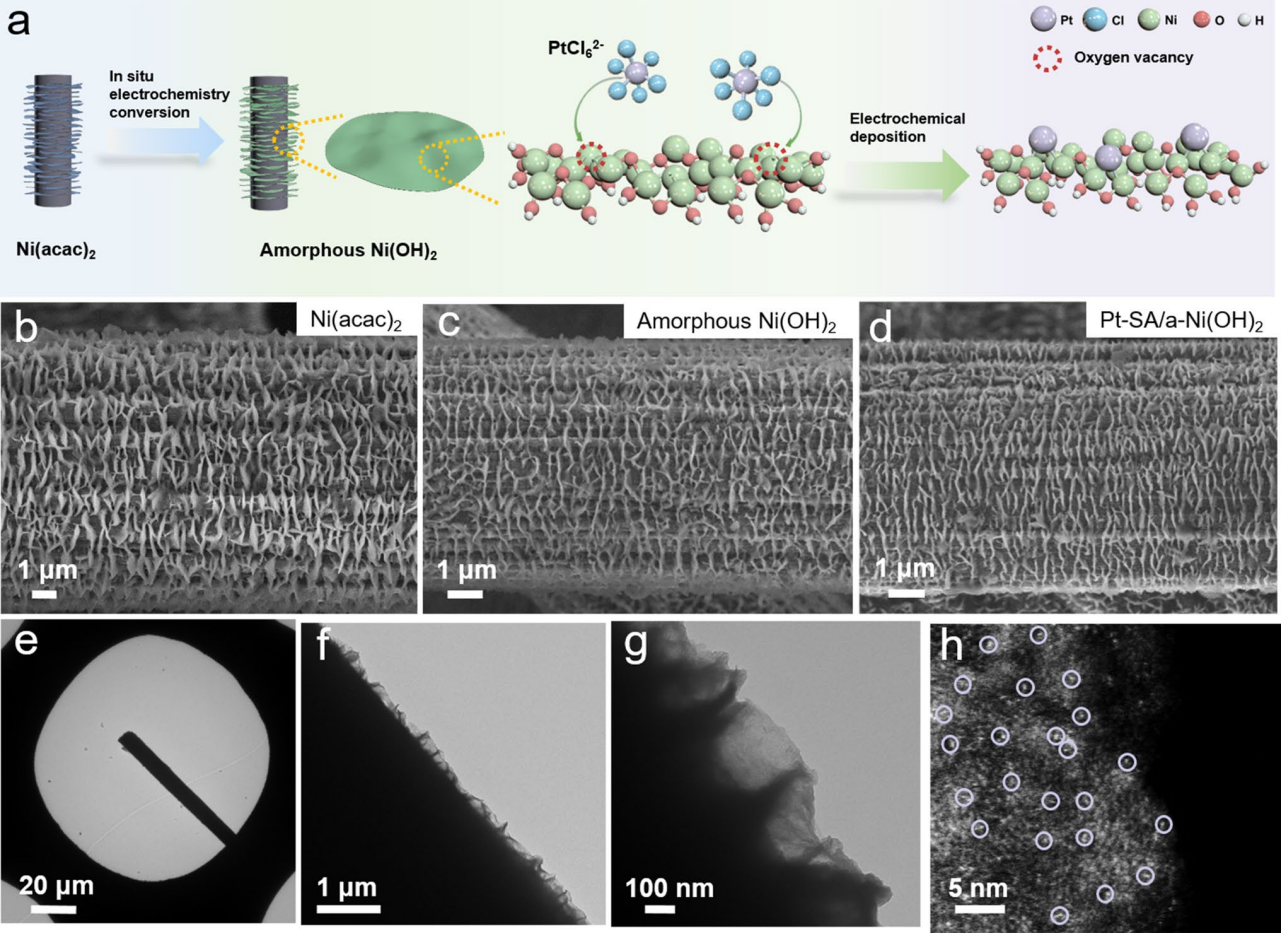
Pt SA on amorphous Ni(OH)_2_ nanosheet arrays. **a** Schematic diagram of Pt–SA/a-Ni(OH)_2_ growth. **b-d** Scanning electron microscopy (SEM) images of Ni(acac)_2_ nanosheet arrays, amorphous Ni(OH)_2_ nanosheet arrays and Pt–SA/a-Ni(OH)_2_ catalyst. **e–g** Step-by-step enlarged TEM images of Pt–SA/a-Ni(OH)_2_ catalyst. **h** AC-HAADF image of Pt–SA/a-Ni(OH)_2_ catalyst

*β*-Ni(OH)_2_ demonstrates a stratified structure connected by Ni–O_6_ octahedrons as units, with hydroxyl groups facing outwards [26]. Thus, when it is employed as the substrate to construct SA catalysts, the loaded metal atoms can only be coordinated with oxygen atoms. In contrast, the amorphous Ni(OH)_2_ presents a large number of unsaturated Ni sites with oxygen vacancies [27], which provide an ideal platform for the establishment of single metal atom bonding to Ni sites (Fig. 1a). The substrate was constructed in two steps. Firstly, Ni(acac)_2_ nanosheet arrays were grown on carbon fibers (Fig. 1b); secondly, amorphous Ni(OH)_2_ nanosheet arrays were generated through the electrochemical transformation from Ni(acac)_2_ nanosheet arrays (Fig. 1c) [28]. Next, Pt SA was loaded on amorphous Ni(OH)_2_ nanosheet arrays (named as Pt–SA/a-Ni(OH)_2_) by electrochemical reduction in 10^−5^ M PtCl_6_^2−^ solution at −0.079 V vs. RHE for 20 min (Fig. 1d). Transmission electron microscopy (TEM) results showed that amorphous Ni(OH)_2_ nanosheets were grown vertically on carbon fibers (Fig. 1e-g), providing a large area for the deposition of Pt SA. Aberration corrected high-angle annular dark field (AC-HAADF) image showed that Ni(OH)_2_ nanosheets have no obvious lattice fringes, evidencing an amorphous structure. The Pt SA as brighter spots are dispersedly loaded on amorphous Ni(OH)_2_ nanosheets, without the appearance of Pt clusters or particles (Fig. 1h). The loading amount of Pt in Pt–SA/a-Ni(OH)_2_ was calculated to be ~ 0.07 wt% through inductively coupled plasma optical emission spectrometer (ICP-OES) (Fig. S1). If the electrochemical reduction time was extended to more than 40 min, Pt nanoparticles (Pt–NP/a-Ni(OH)_2_) were observed (Fig. S2), instead of single atom. The electron paramagnetic resonance (EPR) spectra of the amorphous Ni(OH)_2_ nanosheets and the Pt–SA/a-Ni(OH)_2_ catalyst exhibited typical signals at *g* = 2.003, confirming the existence of the oxygen vacancies in the system (Fig. S3). The presence of oxygen vacancies helps to stabilize Pt SA on the substrate.

### Atomic Structure of Pt–SA/a-Ni(OH)_2_

**Fig. 2:**
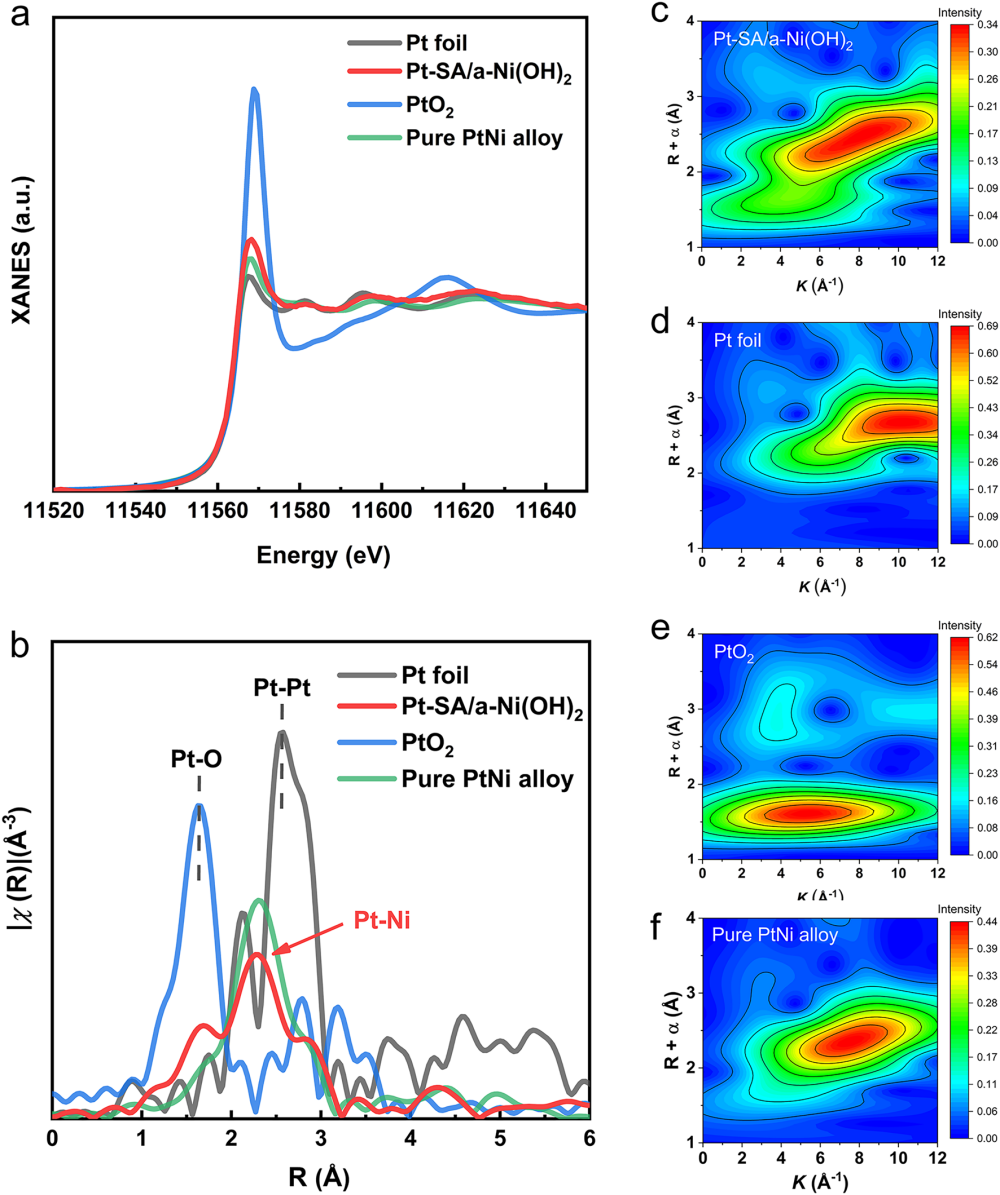
Pt–Ni bonding in Pt–SA/a-Ni(OH)_2_ catalyst. **a** Pt *L*_3_-edge XAFS spectra of Pt–SA/a-Ni(OH)_2_ catalyst, PtO_2_, Pt foil and PtNi alloy. **b** Fourier transformed EXAFS spectra of Pt *L*_3_-edge of Pt–SA/a-Ni(OH)_2_ catalyst, PtO_2_, Pt foil and PtNi alloy. **c-f** Wavelet transform EXAFS of **c** Pt–SA/a-Ni(OH)_2_ catalyst, **d** Pt foil, **e** PtO_2_, **f** PtNi alloy

In order to demonstrate the atomic structure of the Pt–SA/a-Ni(OH)_2_ catalyst, we then investigated the coordination environment of Pt in Pt–SA/a-Ni(OH)_2_ through X-ray absorption fine structure (XAFS) analysis (Fig. 2a). In the Fourier transformed extended X-ray absorption fine structure (EXAFS) spectra (Figs. 2b and S4, Table S1), the Pt foil exhibited a clear Pt–Pt bond at 2.5 Å, and the bond was almost unrecognizable in the Pt–SA/a-Ni(OH)_2_ sample, confirming the single atom state of Pt [29–31].At the same time, the main peak of Pt–SA/a-Ni(OH)_2_ catalyst located at 2.3 Å was also distinguishing from Pt–O bond of standard Pt–O_2_ at 1.6 Å [32, 33]. It meant that Pt–SA/a-Ni(OH)_2_ catalyst displayed a different structure from the conventional SA materials based on Pt–O bonding, which we have also synthesized by employing commercially crystalline Ni(OH)_2_ as a substrate (Fig. S5). It is interesting that the Pt signal in Pt–SA/a-Ni(OH)_2_ is basically the same as that of Pt in the Pt–Ni alloy, which proves the bond of Pt–Ni in Pt–SA/a-Ni(OH)_2_ catalyst (Fig. S6). Similarly, the Pt–Ni bond can be directly observed as the dominant form of Pt present in this system through wavelet transformations [34], rather than the Pt–Pt bond of bulk Pt or the Pt–O bond for conventional Pt SA materials (Fig. 2c-f).

**Fig. 3:**
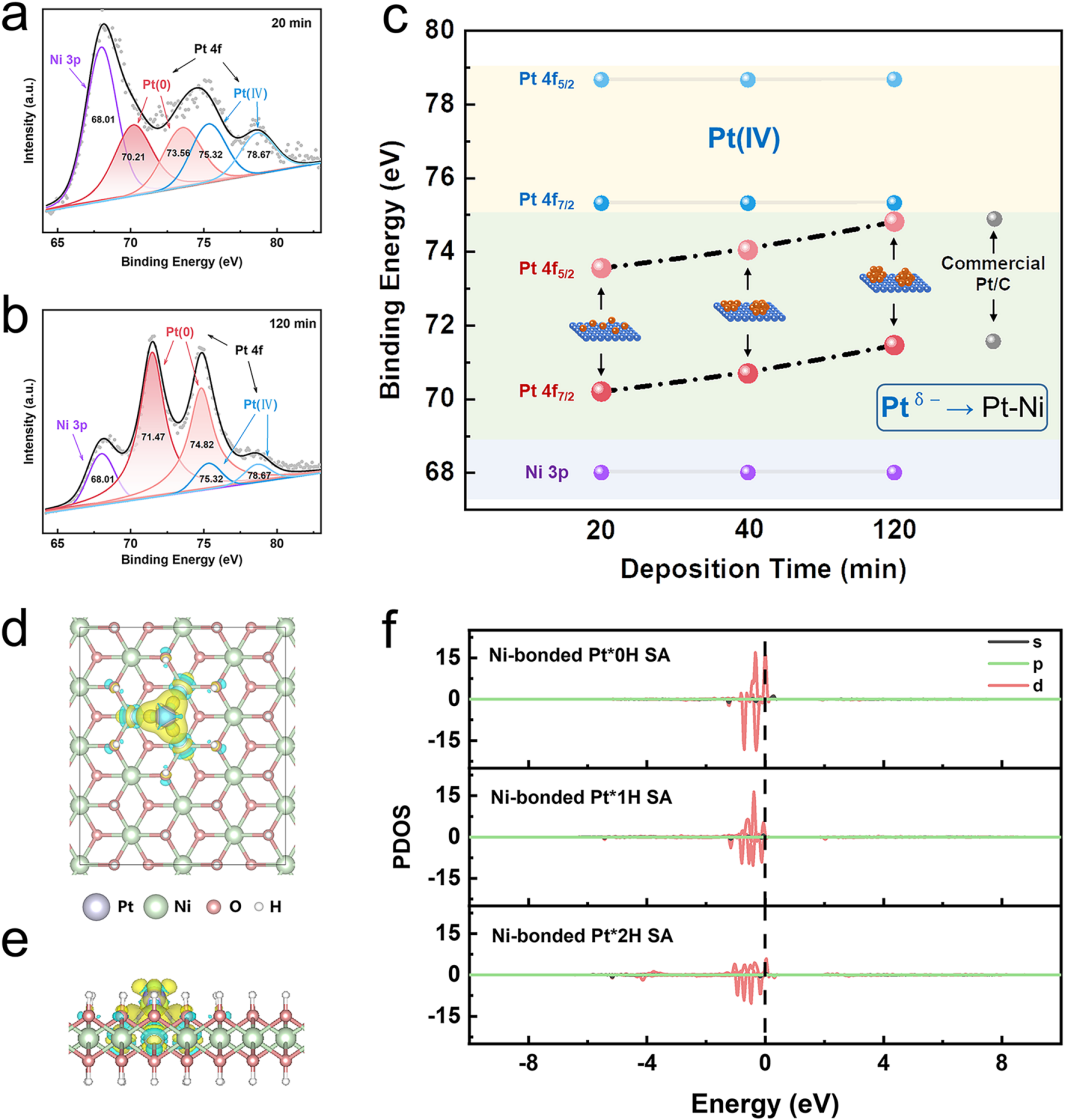
The negatively charged electronic states of Pt^*δ*−^ in Pt–SA/a-Ni(OH)_2_ catalyst. **a, b** Pt 4*f* XPS spectra of Pt/a-Ni(OH)_2_ catalysts with electrodeposition time of 20 and 120 min. **c** Variation of binding energies for Pt/a-Ni(OH)_2_ catalysts with different electrodeposition time. **d, e** Charge density differences of Pt SA adsorption on amorphous Ni(OH)_2_ with oxygen vacancies from top and side views. Isosurfaces are plotted with 0.003 e Å^−3^. The yellow and blue contours represent electron accumulation and depletion, respectively. **f** Calculated partial density of state (PDOS) of Ni-bonded Pt*0H SA, Ni-bonded Pt*1H SA and Ni-bonded Pt*2H SA with the different number of H atoms, and the corresponding number of H adsorption on Pt are 0, 1, and 2 for Ni-bonded Pt*0H SA, Ni-bonded Pt*1H SA, and Ni-bonded Pt*2H SA, respectively

To further verify Pt–Ni bonding in the catalyst, X-ray photoelectron spectroscopy (XPS) characterization was employed to perform series of supported Pt catalysts with different loading forms from Pt SA to Pt nanoparticles, according to the electrodeposition time (20, 40, and 120 min). Two sets of Pt 4*f* peaks were present for Pt(0) and Pt(IV), with an additional Ni 3*p* peak for the Ni(OH)_2_ substrate (Figs. 3a, b and S7). Taking the peak of Ni 3*p* as a reference, the positions of Pt(IV) peak in the catalysts remained unchanged, while that of Pt(0) peak exhibited a regular shift. Interestingly, with the electrodeposition time increasing, the binding energies of Pt 4*f*_7/2_ and Pt 4*f*_5/2_ of Pt(0) gradually shifted to that of commercial Pt/C (Figs. 3c and S8). Thus, the Pt in Pt–SA/a-Ni(OH)_2_ was negatively charged, denoted as Pt^*δ*−^. It indicated that, for Pt–SA/a-Ni(OH)_2_ with the local environment of Pt–Ni bonding, the Pt atom with greater electron-negative can take electrons from Ni atoms and thus became partially negatively charged. When the electrodeposition time increased, Pt species transforms from SA to particle, and the Pt–Pt bonds take advantages, making its valence state closer to 0 valence.

The novel electronic states of Pt SA were further confirmed by DFT calculations. It demonstrated that when Pt SA was adsorbed onto the Ni(OH)_2_ substrate with oxygen vacancies, the three Ni atoms bonded with the Pt SA. As shown in Figs. 3d, e and S9, the charge transfer occurred between Pt SA and the Ni(OH)_2_ substrate, resulting in electron gain by the Pt atoms and manifestation of a negatively charged state, aligning with the experimental XPS results. To unravel the underlying mechanisms for the enhanced HER performance within the Pt SA, we conducted a systematic analysis of the electronic structure of the Pt atom under various hydrogen adsorption scenarios on the Pt SA on Ni(OH)_2_ substrate (Figs. 3f and S10a). When H adsorption on the Pt–SA, the negative charged Pt donates about 0.11 e to first H atom based on the Bader charge, as shown in Table S2. As discussed above, the Pt behaves as negative charge for the whole process. Thus, the electronic structure of Pt–Ni model is greatly different from the counterpart of Pt–O. As shown in Fig. S10b and Table S3, the Pt of Pt–O system tends to accept a small amount of electrons from Ni(OH)_2_ by about −0.03 e. Upon H adsorption, the Pt donates electron to H and Ni(OH)_2_, which makes Pt as positive charge (0.28 e). The calculated density of states (DOS) unveiled that upon hydrogen atoms adsorption onto Pt SA, the extra electrons, donated by the Ni(OH)_2_ substrate to the Pt SA, could transfer to the hydrogen atoms, thus actively promoting the HER process. Consequently, the Ni-bonded Pt SA structure stands as a catalyst with remarkable efficiency in advancing the HER process.

### HER Mechanisms of Pt–SA/a-Ni(OH)_2_ Catalyst

**Fig. 4:**
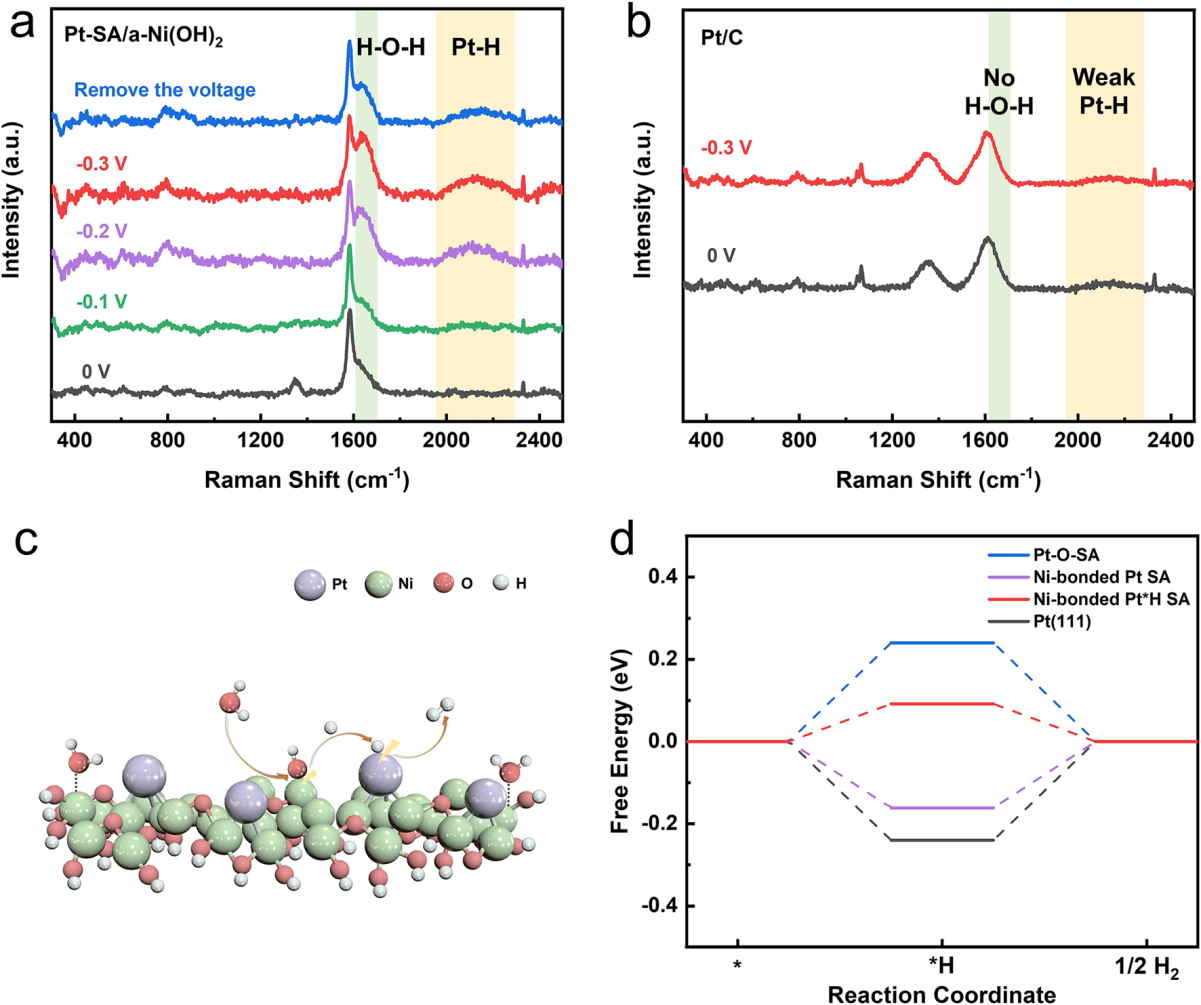
Strong adsorption capacity of hydrogen for the Pt site in Pt–SA/a-Ni(OH)_2_. **a** In situ Raman spectra of Pt–SA/a-Ni(OH)_2_ during HER. **b** In situ Raman spectra of commercial Pt/C during HER. **c** Schematic diagram of the hydrogen spillover processes at Pt–SA/a-Ni(OH)_2_ during HER. **d** Δ*G*_H_ of Pt–O–SA, Ni-bonded Pt SA, Ni-bonded Pt*1H SA and Pt(111). Pt(111) is regarded as a simplified model for Pt NP in Pt/C

Compared to traditional Pt–O bonded Pt SA, Pt–Ni bonded Pt SA has a lower oxidation state, making them more favorable for adsorption of activated hydrogen atoms in the electrolyte, especially under the alkaline [11, 32]. Compared to the widely studied HER in the acidic, the HER under alkaline conditions has milder reaction conditions and higher energy conversion efficiency [35, 36]. However, due to the absence of H^+^ in the solution, the water dissociation step of the HER under alkaline medium is slow and the proton supply is poor, making its rate several orders of magnitude lower than that in acidic medium [37–39]. In order to explore the structural advantages of the Pt–SA/a-Ni(OH)_2_ catalyst for alkaline HER and the possible reaction mechanisms, the adsorbed species of the catalysts during the catalytic reaction was detected by in situ Raman spectroscopy (Fig. S11). As shown in the Raman spectra of Fig. 4a, there is no Pt–H stretching vibration signal at ~ 2170 cm^−1^ before applying voltage [40]. After applying a negative potential for starting HER, a significant Pt–H vibration appeared at ~ 2170 cm^−1^, and the intensity of the peak enhanced synchronously with the increase of the applied negative voltage. It demonstrated the strong adsorption capacity and responsivity of hydrogen for the Pt site in Pt–SA/a-Ni(OH)_2_, consistent with our speculation that Pt–Ni bonding enhances adsorption for hydrogen atoms.

Apart from Pt–H stretching vibration, another additional peak at ~ 1630 cm^−1^ was also observed in Raman spectra when a negative potential was applied. It was attributed to the H–O–H bending vibration [41]. With the increase of the applied negative potential, this peak was also enhanced synchronously, which means that the adsorption of water was also promoted by the catalyst during the HER process. This promotion was also confirmed on pure amorphous Ni(OH)_2_ substrate, demonstrating the enhanced adsorption of H_2_O molecule on amorphous Ni(OH)_2_ (Fig. S12). According to previous report, Ni(OH)_2_ can cleave the H–O–H bond of H_2_O at the interface of Ni(OH)_2_ and Pt, producing hydrogen atoms and transferring them to the active sites for HER reactions [42, 43]. Combined with the synchronously enhanced Pt–H bond, we supposed that amorphous Ni(OH)_2_ can accelerate water dissociation process. The generated H_ad_ intermediates transferred to the Pt SA immediately afterward and thus facilitated HER. To validate this hydrogen spillover process, the in situ Raman spectra of commercial Pt/C in HER was also performed (Fig. 4b) [37, 44]. It was found that the H–O–H bending vibrational signal could not be observed at 1630 cm^−1^, even when a negative voltage was applied. It indicated that compared with amorphous Ni(OH)_2_, the adsorption of water molecules is weak for commercial Pt/C. Meanwhile, almost imperceptible Pt–H stretching vibration was exhibited at ~ 2170 cm^−1^. It suggested that the amorphous Ni(OH)_2_ substrate effectively adsorbed and cleaved water molecules, providing a large number of H_ad_ intermediates for the Pt SA and thus promoting HER (Fig. 4c). Based on the first-principles molecular dynamics (FPMD) simulations, the extra H transfer from Ni(OH)_2_ to Pt was observed for 1.73 ps, indicating that H transfer could occur between Ni(OH)_2_ and Pt for the model of Pt over Ni atom of Ni(OH)_2_, while the H transfer is not observed for the Pt adsorption over the oxygen atom of Ni(OH)_2_ for the same FPMD setups (Fig. S13, Movies S1, S2).

DFT calculations were performed to investigate the connection between the material structures and HER performance, and the calculated Gibbs free energy of hydrogen adsorption (ΔG_H_) were shown in Fig. 4d. The HER performance could be evaluated by the absolute value of ΔG_H_, as proposed by by Nørskov [45]. According to the calculated results, compared with the traditional Pt–O based SA catalysts and Pt NP catalysts, Ni-bonded Pt SA shows the lowest ΔG_H_, indicating the high HER activity (Figs. S14-S16). Furthermore, after the first hydrogen atom has adsorbed on the Pt site, the Δ*G*_H_ of Ni-bonded Pt*H SA for the next H adsorption process decreases to be as small as 0.09 eV (Fig. S14). Therefore, the Ni-bonded Pt SA catalyst could exhibit an excellent catalytic performance of HER.

### HER Performance of Pt–SA/a-Ni(OH)_2_ Catalyst

**Fig. 5:**
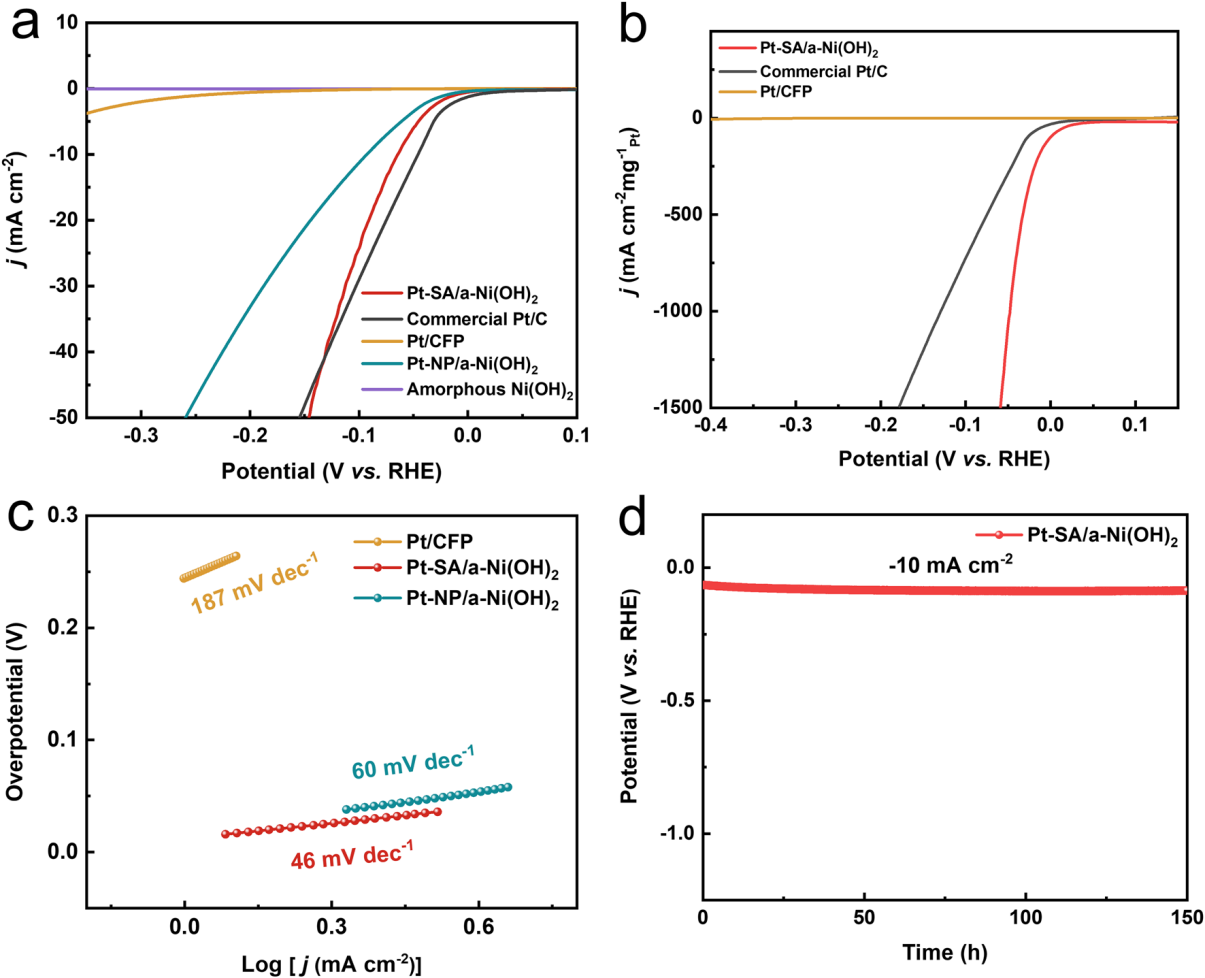
HER performance of Pt–SA/a-Ni(OH)_2_. **a** LSV curves of Pt–SA/a-Ni(OH)_2_ catalyst and the comparative samples. **b** Pt normalized LSV curves of Pt–SA/a-Ni(OH)_2_ catalyst and commercial Pt/C. **c** Tafel slopes of Pt/CFP, Pt–SA/a-Ni(OH)_2_ catalyst, Pt–NP/a-Ni(OH)_2_ catalyst. **d** Stability of Pt–SA/a-Ni(OH)_2_ evaluated through CA test

Then, we quantitatively investigated the alkaline HER performance of Pt–SA/a-Ni(OH)_2_ catalyst. Electrochemical tests were performed in a typical three-electrode system in 1 M KOH, employing carbon rod and Hg/HgO electrode as counter and reference electrode. The HER activity of the Pt–SA/a-Ni(OH)_2_ catalyst was firstly evaluated by analyzing linear sweep voltammetry (LSV) curves. To reach a current density of −10 mA cm^−2^ in alkaline solution, Pt–SA/a-Ni(OH)_2_ catalyst with ultra-low noble metal loading (~ 0.07 wt%) exhibited an overpotential of only 64 mV (Fig. 5a). This overpotential is significantly lower than that of the a-Ni(OH)_2_ substrate and the other comparative samples with different types of Pt-based bonds (Figs. 5a and S17), which is also comparable to some recently reported Pt based works (Table S4) [5, 8, 46–49]. Furthermore, normalized the mass of Pt, its overpotential is only 48 mV at −1000 mA cm^−2^ mg^−1^_Pt_, which is considerably lower than the overpotential of commercial Pt/C of 128 mV (Figs. 5b and S18). The remarkable performance should be attributed to the above mentioned SA of Pt–Ni bonding configuration and its synergistic effect with the amorphous substrates. To confirm the HER mechanism, we conducted the kinetic process of the reaction through calculating the value of Tafel slope (Fig. 5c). The Tafel slope of 46 mV dec^−1^ for Pt–SA/a-Ni(OH)_2_ is much lower than those of Pt/CFP and Pt–NP/a-Ni(OH)_2_, indicating that the Volmer step of water dissociation in alkaline HER was accelerated in the Pt–SA/a-Ni(OH)_2_, in agreement with the Raman spectra results. In addition, the durability of Pt–SA/a-Ni(OH)_2_ catalyst was also evaluated through chronoamperometry (CA) test, which showed that the catalyst kept excellent stability during 150 h of HER (Fig. 5d). The morphology and atomic structure were also well maintained (Fig. S19).

## Conclusion

In summary, utilizing the exposed Ni sites with oxygen vacancies on amorphous Ni(OH)_2_ to anchor Pt, Pt–Ni bonding fixed Pt SA are constructed, instead of Pt–O bonding in the conventional Pt SA catalysts. The novel structure greatly facilitates alkaline HER process by both the unique atomic and electronic structures. The amorphous Ni(OH)_2_ substrate effectively adsorbs and cleaves water molecules, accelerating the Volmer step of water dissociation in alkaline HER and providing H_ad_ for the Pt site. Furthermore, the Pt–Ni bond reduces the oxidation state of the Pt SA and thus optimize the adsorption capacity for hydrogen atoms. This work provides a new idea to deeply modulate the configuration of SA catalysts and thus controllably regulate the electronic states of SA catalysts to enhance catalytic performance.

## Supplementary Information

Below is the link to the electronic supplementary material.

Supplementary file1 (MP4 2346 kb)

Supplementary file2 (MP4 2649 kb)

Supplementary file3 (DOCX 5750 kb)
