# Supplementary material for: Achieving Negatively Charged Pt Single Atoms on Amorphous Ni(OH)2 Nanosheets with Promoted Hydrogen Absorption in Hydrogen Evolution
Source: Nanomicro Lett. 2024 May 23;16:202. doi: 10.1007/s40820-024-01420-6 (PMC11116366; doi:10.1007/s40820-024-01420-6)
Supplement: Supplementary file 3 — Supplementary file3 (DOCX 5750 kb) [file 40820_2024_1420_MOESM3_ESM.docx]

Supporting Information for

**Achieving Negatively Charged Pt Single Atoms on Amorphous Ni(OH)_2_ Nanosheets with Promoted Hydrogen Absorption in Hydrogen Evolution**

Yue Liu^1,2,†^, Gui Liu^1,†^, Xiangyu Chen^1,†^, Chuang Xue^1^, Mingke Sun^1^, Yifei Liu^1^, Jianxin Kang^1,^*, Xiujuan Sun^2,^* and Lin Guo^1,^*

^1^School of Chemistry, Beijing Advanced Innovation Center for Biomedical Engineering, Key Laboratory of Bio-Inspired Smart Interfacial Science and Technology, Beihang University, Beijing 100191, P. R. China

^2^School of Chemistry, Key Laboratory of Environmentally Friendly Chemistry and Applications of Ministry of Education, Xiangtan University, Hunan 411105, P. R. China

^†^Yue Liu, Gui Liu, and Xiangyu Chen contributed equally to this work.

*Corresponding authors. E-mail: kang[jianxin@buaa.edu.cn](mailto:jianxin@buaa.edu.cn) (Jianxin Kang), [sunxj594@xtu.edu.cn](mailto:sunxj594@xtu.edu.cn) (Xiujuan Sun); [guolin@buaa.edu.cn](mailto:guolin@buaa.edu.cn) (Lin Guo)

**S1 Electrochemical Experiment**

In a typical three-electrode configuration cell, electrochemical tests were carried out on a CHI 660e electrochemical workstation (Shanghai Chenhua, China) and WaveDriver 200 (PINE, USA) with the prepared sample as the working electrode and the carbon rod and the Hg/HgO (1 M KOH solution) electrode as the counter electrode and reference electrode, respectively. The electrolyte for HER was 1 M KOH, pH = 13.7. Electrochemical measurements of all samples were carried out under the same test conditions. Oxygen was expelled by passing argon gas through the electrolyte for 30 minutes prior to testing, and then electrochemical measurements were performed in an environment where argon gas was passed through all the time. The potential herein is referenced to the reversible hydrogen electrode (RHE) with the following equation: E_(RHE)_ = E_(Hg/HgO)_ + 0.098 + 0.059 × pH. LSV curves were used to measure the electrocatalytic activity of the catalyst over a potential range of -0.479 ~ 0.221 V *vs.* RHE at a scan rate of 5 mV s^-1^, with all polarisation curves were 90% iR-compensated, where i is the current and R is the uncompensated electrolyte ohmic resistance as measured by electrochemical impedance spectroscopy (EIS). The current density was calculated from the actual area of the prepared catalyst immersed in the electrolyte. According to the Tafel equation: *η* = *a* + *b*log *j*, using the logarithm of the current density as the X-axis and the overpotential as the Y-axis, a Tafel plot is obtained, where *a* is the Tafel constant, *b* is the Tafel slope, *j* is the cathodic current density, and *η* is the overpotential.

**S2 Computational Method**

The calculations were carried out using DFT, with the Vienna ab-initio simulation package (VASP) [S1, S2]. The exchange-correlations functional was described by a generalized gradient approximation of Perdew-Burke-Ernzerhof (GGA-PBE) [S3]. On-site Hubbard correction U of Ni-3d orbits was set to 5.5 eV. The energy cutoff for the plane wave was 400 eV. A 15 Å vacuum was used. The Fermi scheme was employed for electron occupancy with an energy smearing of 0.1 eV. The first Brillouin zone was sampled using a 2 × 2 × 1 point grid by the Monkhorst-Pack K-points scheme [S4]. The DFT-D3 method was used to calculate the van der Waals interactions [S5]. The energy (converged to 1.0 ×10^-5^ eV/atom) and force (converged to 0.01 eV/Å) were set as the convergence criterion for geometry optimization. The spin polarization was considered in all calculation.

For HER, the Computational Hydrogen Electrode model (CHE) [S6] employs the Gibbs free-energy change curves to estimate the activity of the catalyst. Free energy change from initial states to final states of the reaction was calculated as follows [S7]:

$$\Delta G=\Delta E+\Delta ZPE-T\Delta S$$

Where *ΔE* is the total energy change based on the DFT calculations, *ΔZPE* and *ΔS* are the change in the zero-point energy and the entropy, respectively, *T* is room temperature (298.15 K). The free energy of (H^+^+e^-^) at standard conditions was assumed as the energy of 1/2 H_2_. The entropies of the HER intermediates were calculated from the vibrational frequencies.

**S3 Supplementary Figures**


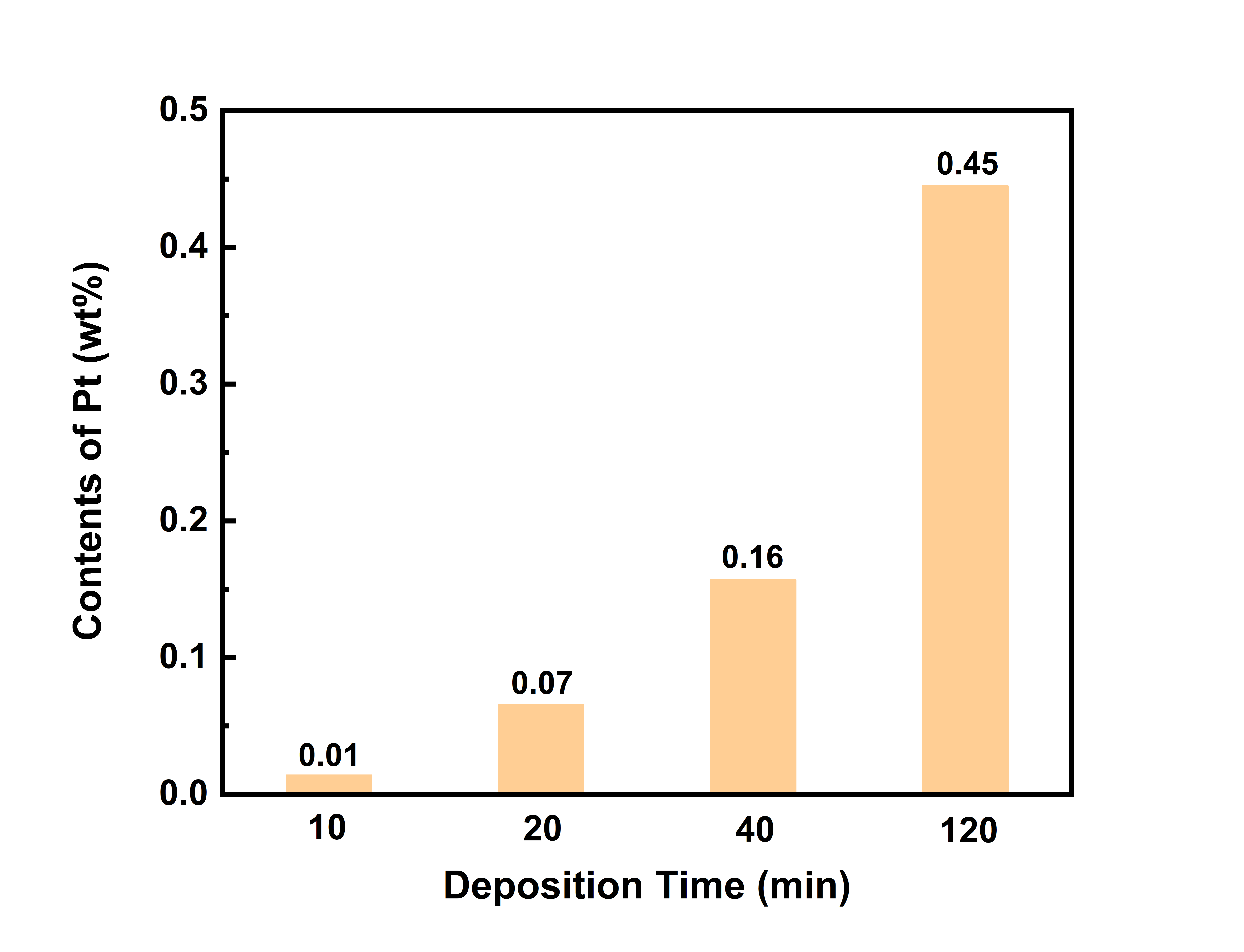


**Fig. S1** Pt contents in as-prepared catalysts with varied Pt deposition time (10 min, 20 min, 40 min, 120 min) through ICP-OES


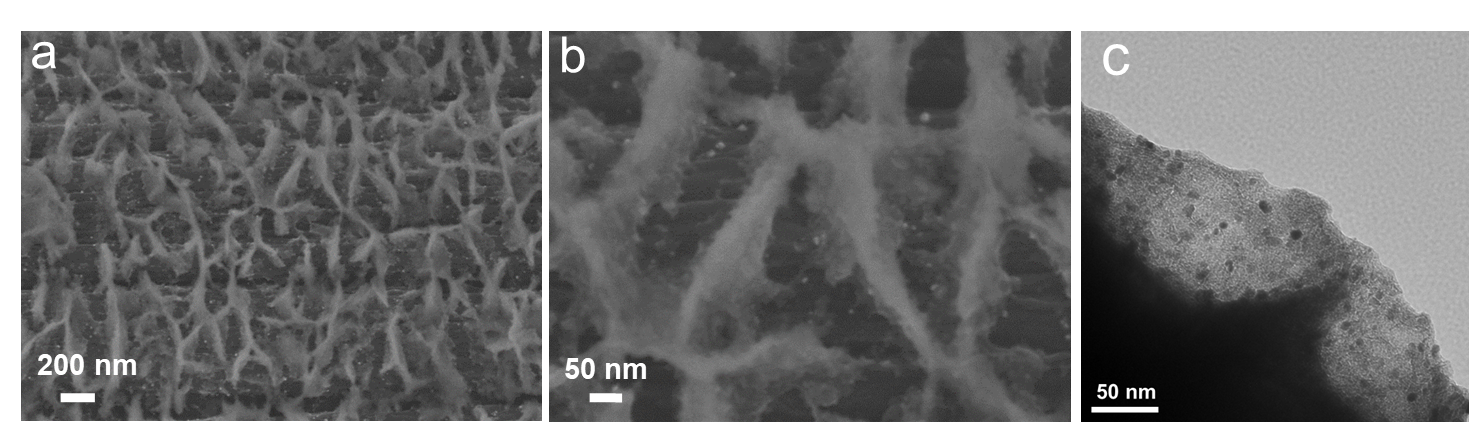


**Fig. S2 a, b** SEM images of Pt-NP/a-Ni(OH)_2_ at varied magnifications. **c** TEM images of Pt-NP/a-Ni(OH)_2_, in which Pt nanoparticles can be clearly observed


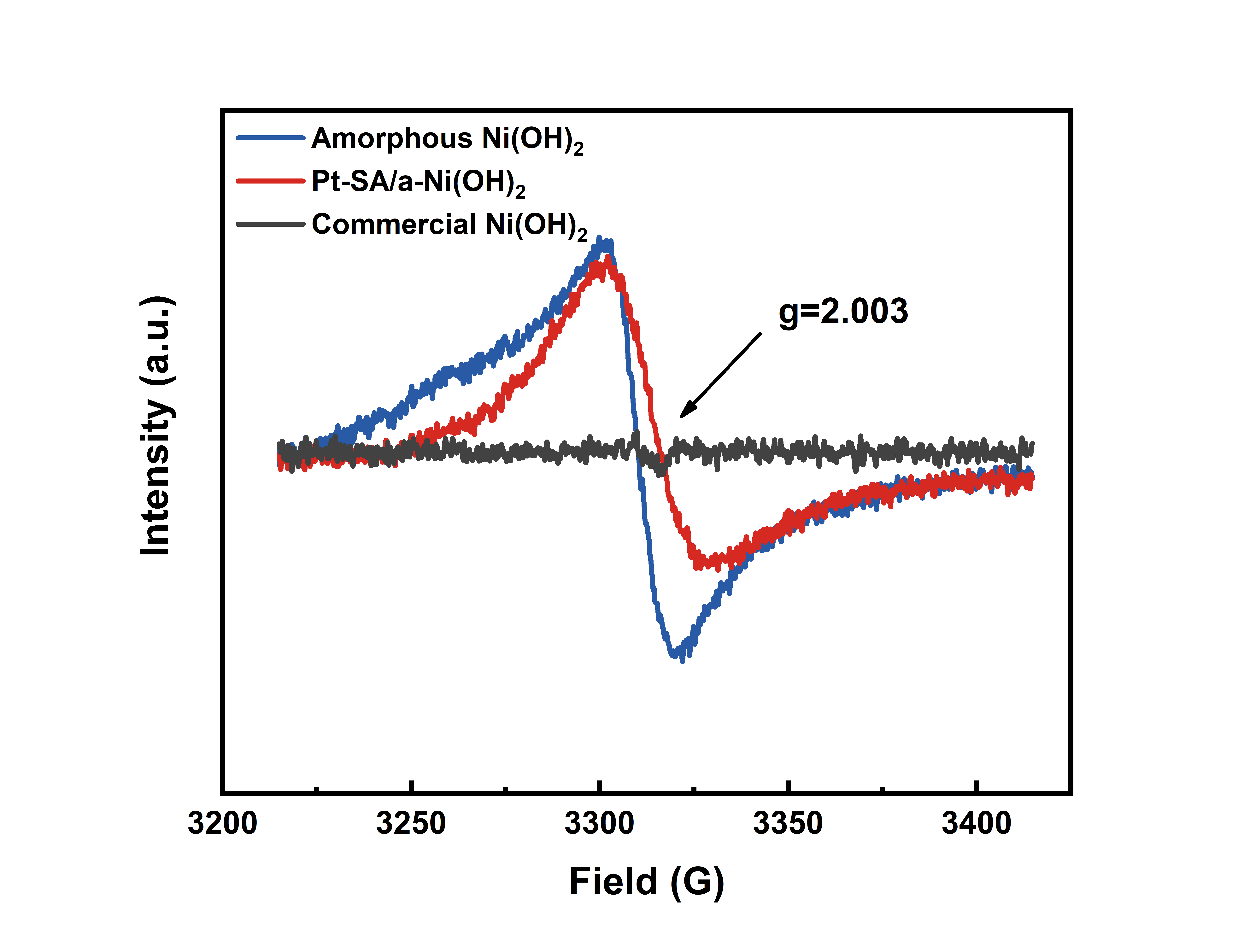


**Fig. S3** EPR spectra of the amorphous Ni(OH)_2_, Pt-SA/a-Ni(OH)_2_ and commercial Ni(OH)_2_.


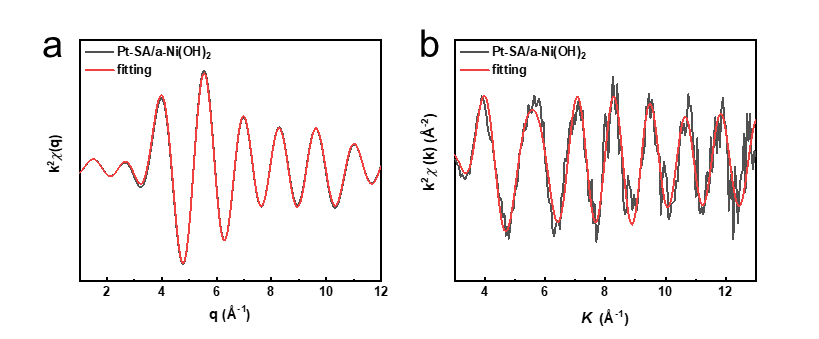


**Fig. S4 a** q space fitting curve at Pt *L*_3_-edge of Pt-SA/a-Ni(OH)_2_. **b** k space fitting curve at Pt *L*_3_-edge of Pt-SA/a-Ni(OH)_2_


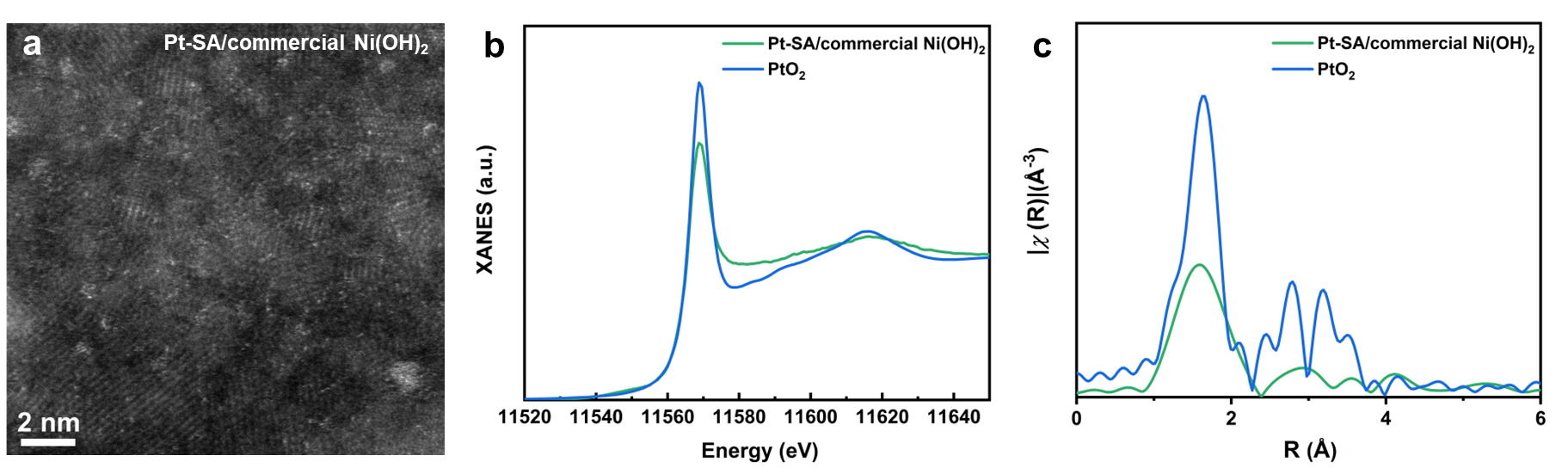


**Fig. S5 a** HAADF-STEM image of Pt-SA/commercial Ni(OH)_2_ catalyst. **b** The Pt *L*_3_-edge XANES spectra of Pt-SA/commercial Ni(OH)_2_ and PtO_2_. **c** Fourier transformed EXAFS spectra of Pt *L*_3_-edge of Pt-SA/commercial Ni(OH)_2_ and PtO_2_

_
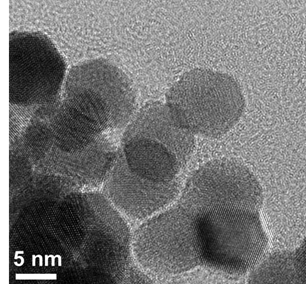
_

**Fig. S6** TEM image of the as-prepared PtNi alloy


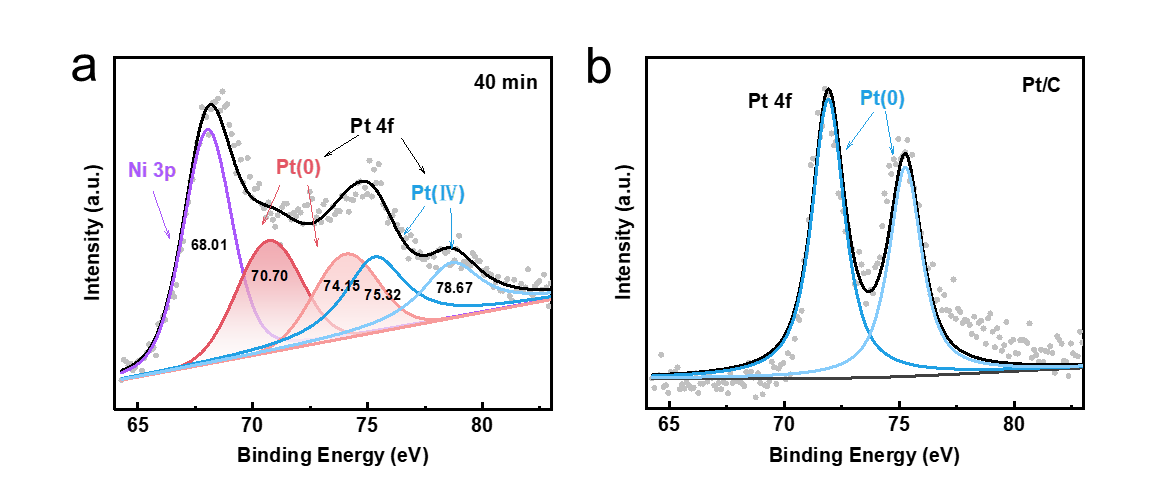
**Fig. S7 a** Pt 4*f* XPS spectra of Pt/a-Ni(OH)_2_ catalyst with electrodeposition time of 40 min. **b** Pt 4*f* XPS spectra of Pt/C catalyst

**Fig. S8** The Pt 4*f* XPS spectra of Pt/a-Ni(OH)_2_ catalyst with electrodeposition time of 5 min. It could be found that the regularity of red shifted Pt(0) is further enhanced


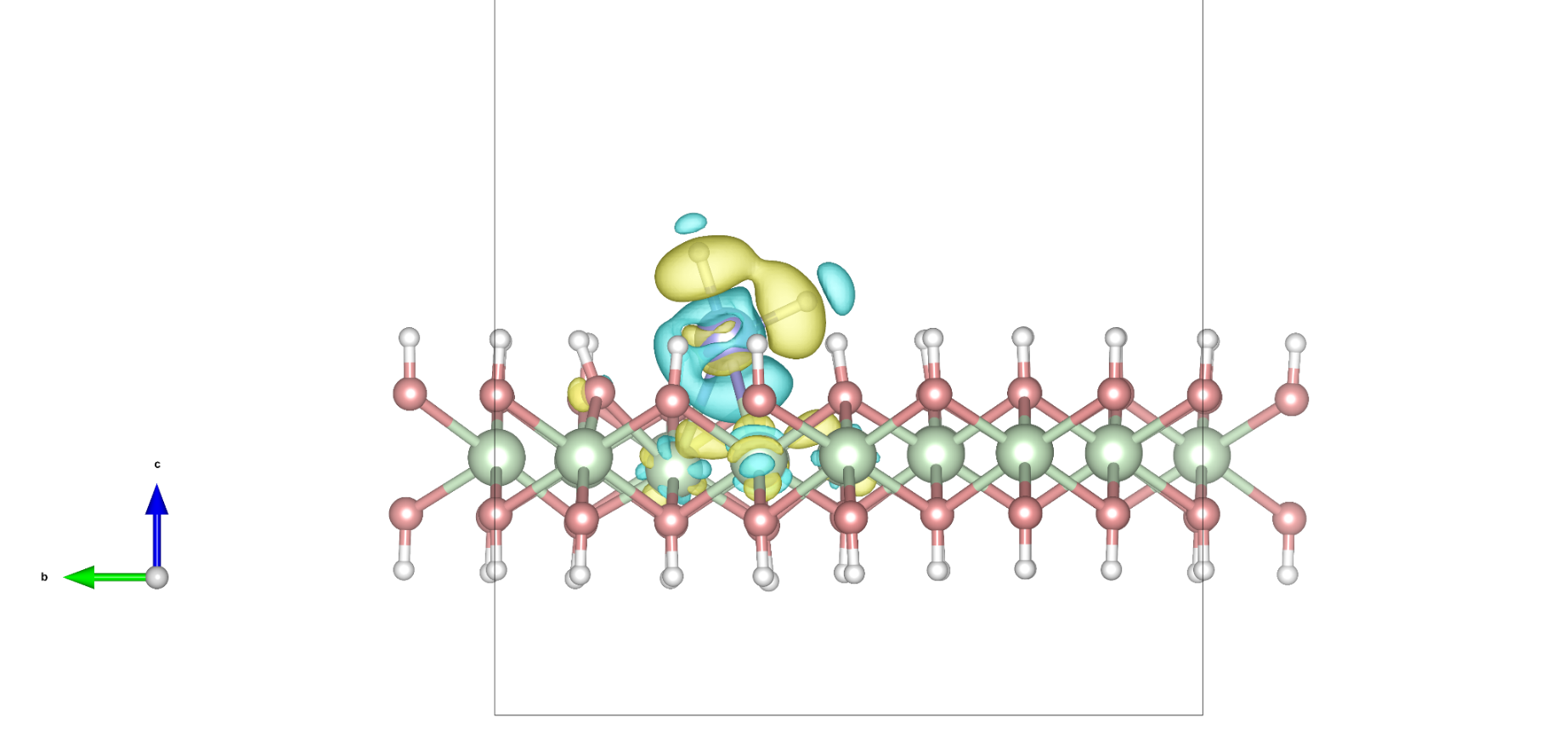


**Fig. S9** Calculated charge density differences of two H atoms adsorbed on Pt-SA/a-Ni(OH)_2_ (isosurfaces = 0.003 e/Å^3^), and the yellow and blue contours represent electron accumulation and depletion, respectively. Here, purple, green, red and white balls represent the Pt, Ni, O and H atoms, respectively


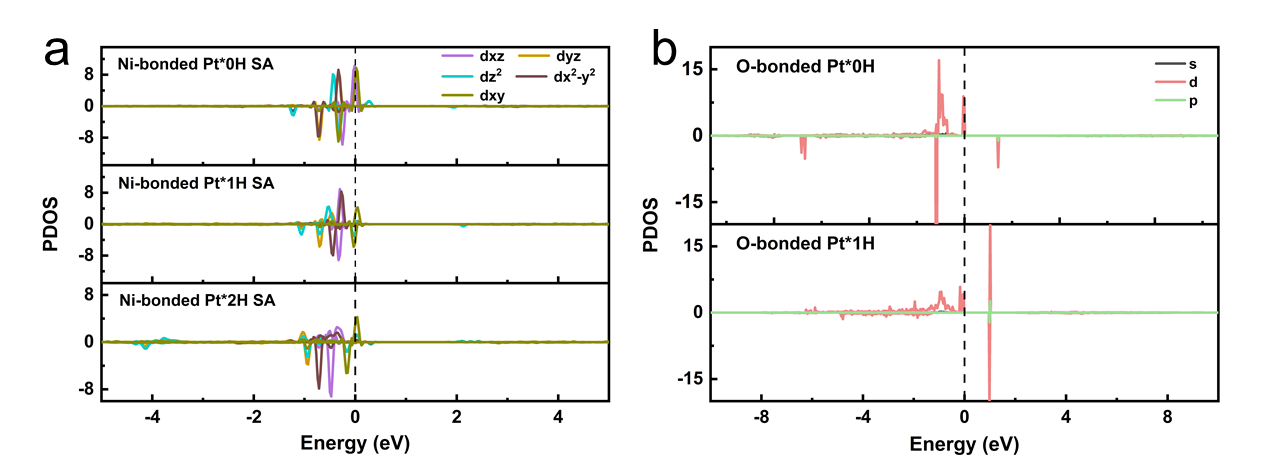


**Fig. S10 a** The PDOS diagrams of Pt atoms for Ni-bonded Pt with different amount of H atoms. Upper panel: without H adsorption (Pt*0H SA); Middle panel: one H adsorption (Pt*1H SA), lower panel: two H adsorption (Pt*2H SA). **b** The PDOS diagrams of Pt atoms for O-bonded Pt*0H and O-bonded Pt*1H


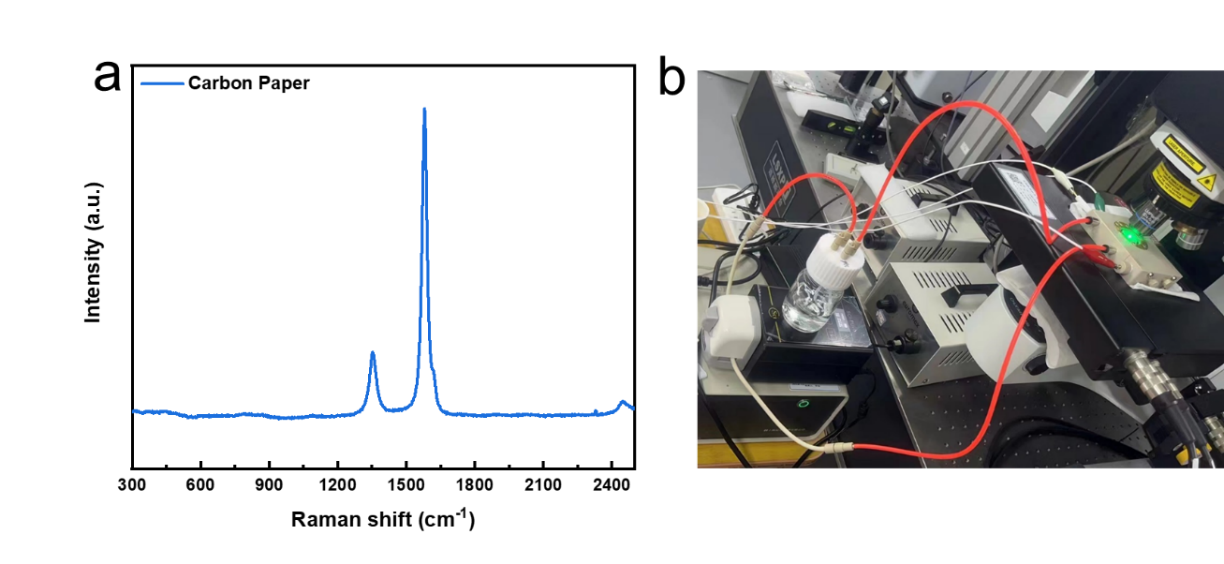


**Fig. S11 a** Raman spectra of CFP. **b** Photo of the *in situ* electrochemical Raman testing

**Fig. S12** *In situ* Raman spectra of amorphous Ni(OH)_2_ during HER


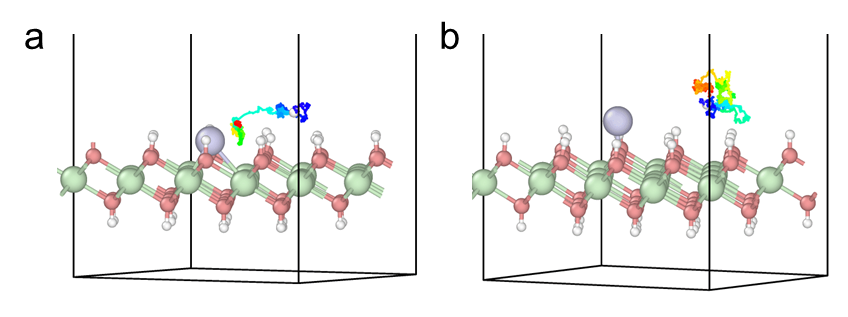


**Fig. S13** The H transfer behavior between Pt and Ni(OH)_2_ of Pt-Ni model (**a**) and Pt-O model (**b**) based on first-principles molecular dynamics (FPMD) simulations for 10 ps. The trajectory of the H atom as a function of time were shown in line by different colors The initial part is shown in blue, while the final stage was shown in red. Red, white, green and purple balls stand for O, H, Ni, and Pt atoms, respectively


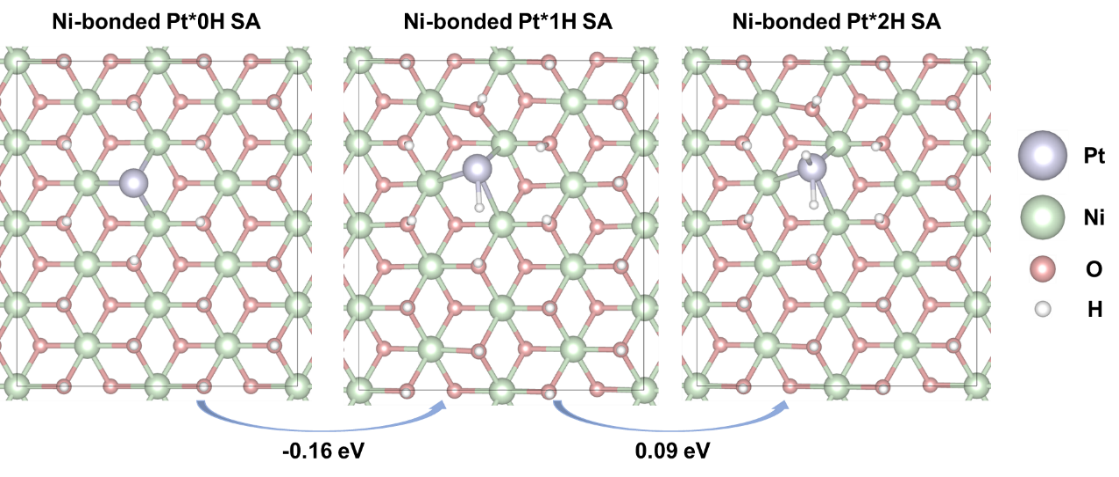


**Fig. S14** The typical configurations of Ni-bonded Pt with different amount of H atoms. Left panel: without H adsorption (Pt*0H SA); middle panel: one H adsorption (Pt*1H SA); right panel: two H adsorption (Pt*2H SA). Here, purple, green, red and white balls represent the Pt, Ni, O and H atoms, respectively


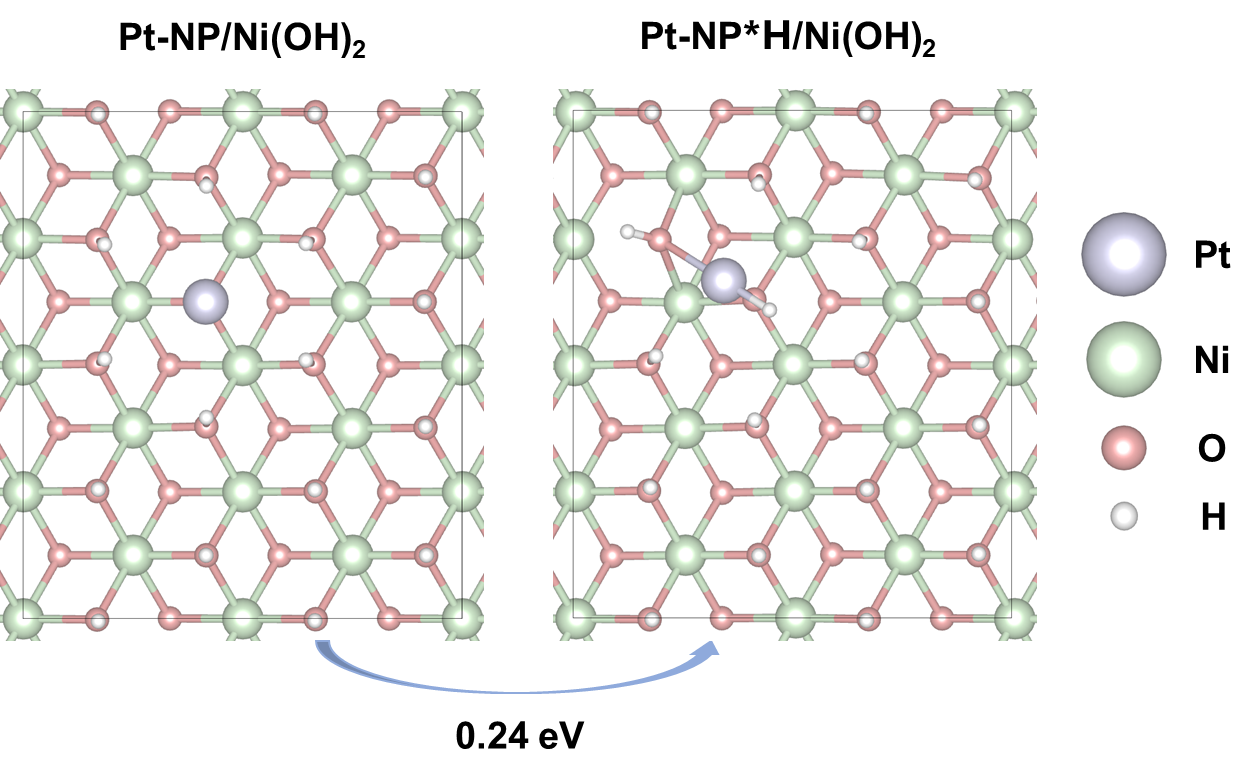


**Fig. S15** The atomic model of Pt-NP/Ni(OH)_2_, and Pt-NP*H/Ni(OH)_2_. Here, purple, green, red and white balls represent the Pt, Ni, O and H atoms, respectively

**
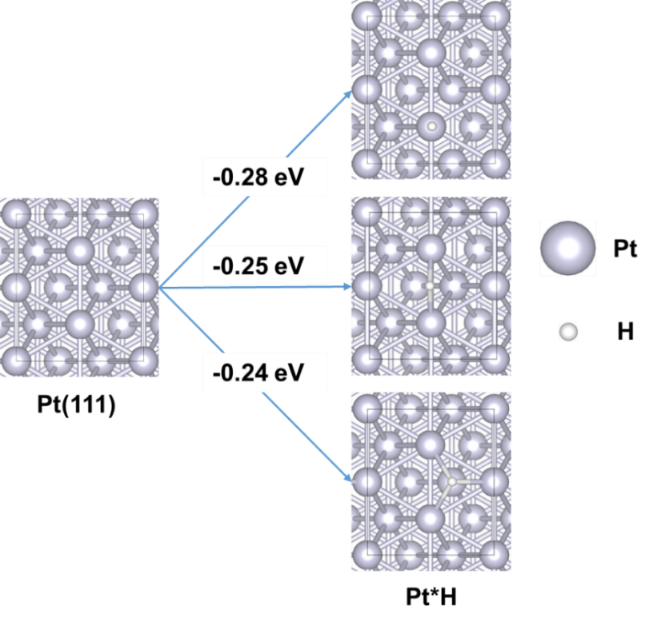
**

**Fig. S16** The atomic model of different configurations for Pt(111) adsorption hydrogen atoms. Here, purple and white balls represent the Pt and H atoms, respectively


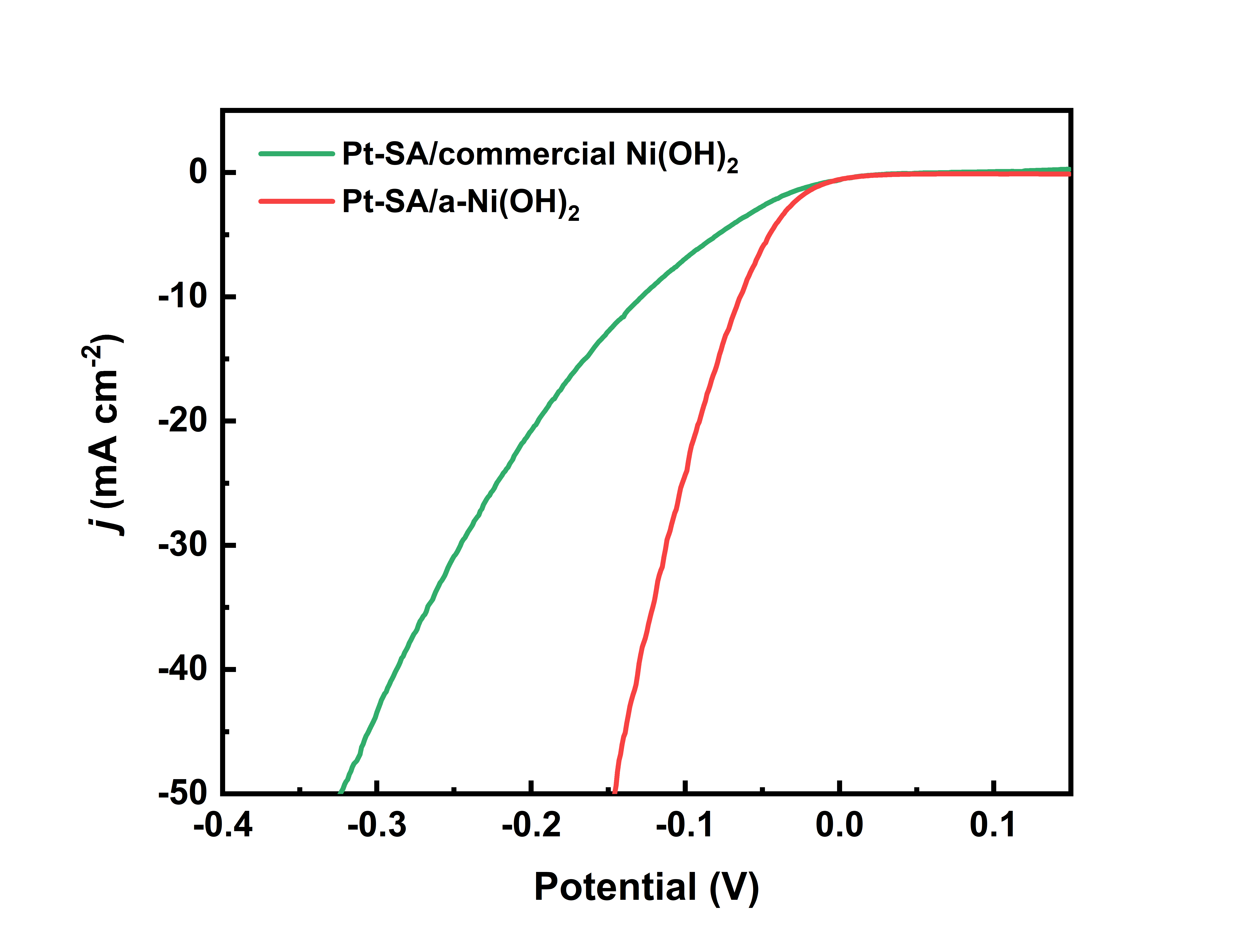


**Fig. S17** LSV curves of Pt-SA/a-Ni(OH)_2_ catalyst and Pt-SA/commercial Ni(OH)_2_


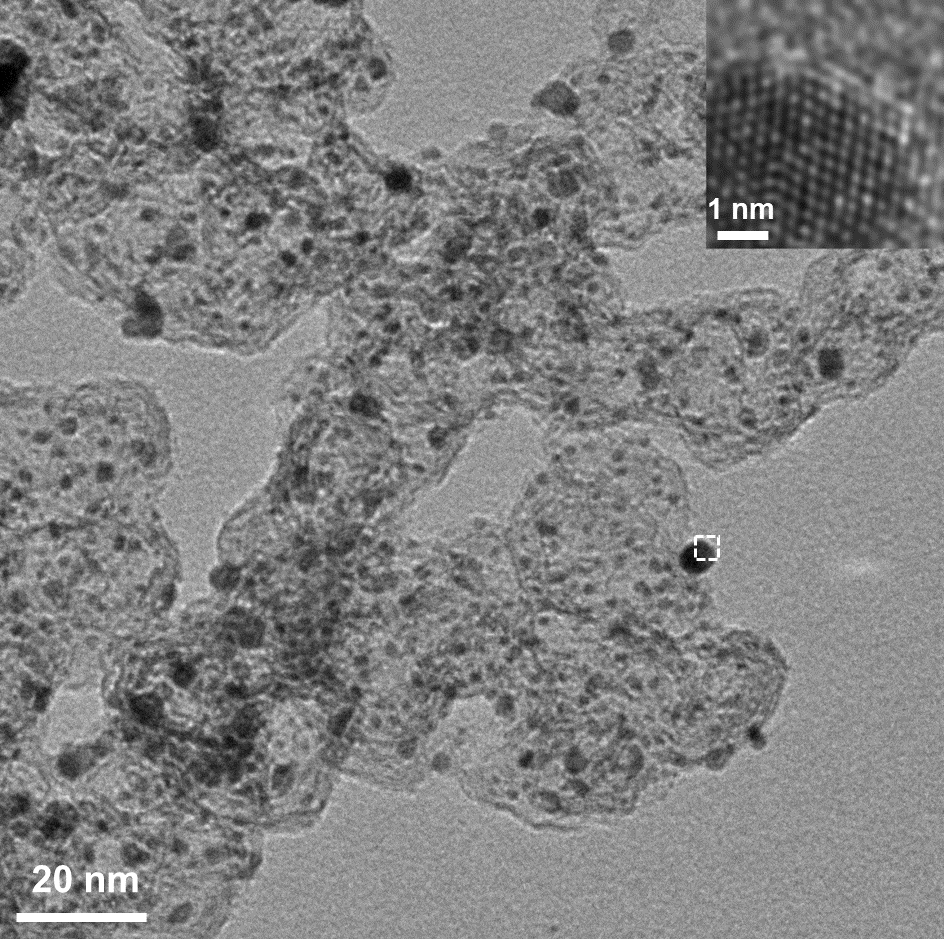


**Fig. S18** TEM image of Pt/C. The inset shows the high-resolution transmission image labeled by the white frame. Both the atomic arrangement and the lattice spacing are consistent with Pt(111)


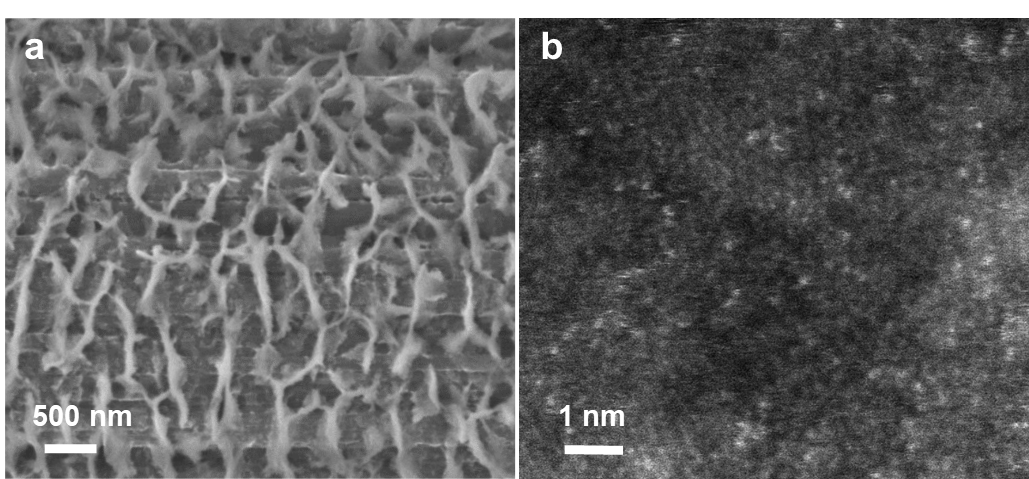


**Fig. S19** **a** SEM image of Pt-SA/a-Ni(OH)_2_ stabilized after test. **b** HAADF image of Pt-SA/a-Ni(OH)_2_ stabilized after test

**S4 Supplementary Tables**

**Table S1** Structural parameters extracted from the Pt L_3_-edge EXAFS fitting. (S_0_^2^=0.89)

| **sample** | **Scattering pair** | **CN** | **R (Å)** | **σ^2^ (10^-3^Å^2^)** | **ΔE_0_ (eV)** | **R factor** |
| --- | --- | --- | --- | --- | --- | --- |
| Pt-SA/a-Ni(OH)_2_ | Pt-O | 1.2 | 1.98 | 7.3 | 6.6 | 0.006 |
|  | Pt-Ni | 8.2 | 2.65 | 6.7 |  | 0.004 |

**Table S2** Calculated Bader charge differences between the isolated Pt and the Pt of Pt-Ni model without and with H adsorption. As for H adsorption, both one and two H atoms were considered, respectively

|  | Pt-SA/a-Ni(OH)_2_ | Pt*H-SA/a-Ni(OH)_2_ | Pt*2H-SA/a-Ni(OH)_2_ |
| --- | --- | --- | --- |
| Pt | -0.58 | -0.47 | -0.31 |
| The first H atom | - | -0.11 | -0.12 |
| The second H atom | - | - | -0.06 |

**Table S3** Calculated Bader charge difference between the isolated Pt and the Pt of Pt-O model-SA/a-Ni(OH)_2_ without and with H adsorption

|  | Pt-O SA | Pt*H-O SA |
| --- | --- | --- |
| Pt atom | -0.03 | 0.28 |
| Adsorbed H atom | * | -0.12 |

**Table S4** Comparison of the electrocatalytic activity of Pt-SA/a-Ni(OH)_2_ for HER with catalysts from previous reports

| Electrocatalysis | Overpotential (10 mA cm^-2^) | electrolytes | Content of Pt | References |
| --- | --- | --- | --- | --- |
| Pt-SA/a-Ni(OH)_2_ | 64 mV | 1 M KOH | 0.07 wt% | This work |
| Pt@PCM | 139 mV | 1 M KOH | 0.53 wt% | *Sci. Adv.*, 2018, 4(1), eaao6657 |
| Pt/Nb-Co(OH)_2_ | 112 mV | 1 M KOH | 3.34 wt% | *Small*, 2023, 19(20), 2207569 |
| Pt-Ni(OH)_x_ | 58 mV | 1 M KOH | 0.17 wt% | *Adv. Energy Mater.*, 2023, 13(10), 2203955 |
| Pt_5_/HMCS | 46.2 mV | 1 M KOH | 5.08 wt%. | *Adv. Mater.*,2020, 32(7), 1901349 |
| PtSA-NiSe-V | 45 mV | 1 M KOH | 3.2 wt% | *Angew. Chem. Int. Ed*, 2023, 62(39), e202308686 |
| Pt@DG | 37 mV | 1 M KOH | 1.57 wt% | *J. Am. Chem. Soc.*, 2022, 144(5), 2171–2178 |

**Supplementary References**

1. G. Kresse, J. Furthmüller, Efficiency of ab-initio total energy calculations for metals and semiconductors using a plane-wave basis set. Comput. Mater. Sci. **6**(1), 15-50 (1996). <https://doi.org/10.1016/0927-0256(96)00008-0>
2. G. Kresse, J. Furthmüller, Efficient iterative schemes for ab initio total-energy calculations using a plane-wave basis set. Phys. Rev. B. **54**(16), 11169-11186 (1996). <https://doi.org/10.1103/PhysRevB.54.11169>
3. J.P. Perdew, J.A. Chevary, S.H. Vosko, K.A. Jackson, M.R. Pederson et al., Erratum: Atoms, molecules, solids, and surfaces: Applications of the generalized gradient approximation for exchange and correlation. Phys. Rev. B. **48**(7), 4978-4978 (1993). <https://doi.org/10.1103/PhysRevB.48.4978.2>
4. H.J. Monkhorst, J.D. Pack, Special points for Brillouin-zone integrations. Phys. Rev. B. **13**(12), 5188-5192 (1976). <https://doi.org/10.1103/PhysRevB.13.5188>
5. S. Grimme, J. Antony, S. Ehrlich, H. Krieg, A consistent and accurate ab initio parametrization of density functional dispersion correction (DFT-D) for the 94 elements H-Pu. J. Chem. Phys. **132**(15), 154104 (2010). <https://doi.org/10.1063/1.3382344>
6. J.K. Nørskov, T. Bligaard, A. Logadottir, J.R. Kitchin, J.G. Chen et al., Origin of the Overpotential for Oxygen Reduction at a Fuel-Cell Cathode. J. Phys. Chem. B. **108**(46), 17886-17892 (2004). <https://doi.org/10.1021/jp047349j>
7. J.K. Nørskov, T. Bligaard, A. Logadottir, J.R. Kitchin, J.G. Chen et al., Trends in the Exchange Current for Hydrogen Evolution. J. Electrochem. Soc. **152**(3), J23 (2005). <https://doi.org/10.1149/1.1856988>
